# Curcumin downregulates the expression of Snail via suppressing Smad2 pathway to inhibit TGF-β1-induced epithelial-mesenchymal transitions in hepatoma cells

**DOI:** 10.18632/oncotarget.22590

**Published:** 2017-11-21

**Authors:** Meng-Ting Cao, Hui-Fang Liu, Zhi-Gang Liu, Ping Xiao, Jing-Jing Chen, Yuan Tan, Xiao-Xin Jiang, Zhi-Chao Jiang, Yu Qiu, Hong-Jun Huang, Qiu-Gui Zhang, Guan-Min Jiang

**Affiliations:** ^1^ Department of Clinical Laboratory, Hunan Cancer Hospital and The Affiliated Cancer Hospital of Xiangya School of Medicine, Central South University, Changsha, Hunan, China; ^2^ Department of Clinical Laboratory, The First Affiliated Hospital of University of South China, Hengyang, Hunan, China; ^3^ Department of Radiation Oncology, Hunan Cancer Hospital and The Affiliated Cancer Hospital of Xiangya School of Medicine, Central South University, Changsha, Hunan, China; ^4^ Sinocare Biosensing Limited Company, Changsha, Hunan, China; ^5^ Department of Integrated Traditional Chinese and Western Medicine, The Second People’s Hospital of Hunan Province, Changsha, Hunan, China; ^6^ Department of ICU, Hunan Children's Hospital, Changsha, Hunan, China

**Keywords:** curcumin, epithelial-mesenchymal transitions, transcriptional activation, snail, tumor metastasis

## Abstract

Hepatocellular carcinoma (HCC) remains the third cause of cancer-related mortality. Resection and transplantation are the only curative treatments available but are greatly hampered by high recurrence rates and development of metastasis, the initiation of cancer metastasis requires migration and invasion of cells, which is enabled by epithelial-mesenchymal transitions (EMT). TGF-β1 is a secreted protein that performs many cellular functions, including the control of cell growth, cell proliferation, cell differentiation and apoptosis. TGF-β1 is known as a major inducer of EMT, and it was reported that TGF-β1 induced EMT via Smad-dependent and Smad-independent pathways. However, the extrinsic signals of TGF-β1 regulated the EMT in hepatoma cells remains to be elucidated, and searching drugs to inhibit TGF-β1 induced EMT may be considered to be a potentially effective therapeutic strategy in HCC. Fortunately, in this study, we found that curcumin inhibited TGF-β1-induced EMT in hepatoma cells. Furthermore, we demonstrated that curcumin inhibited TGF-β1-induced EMT via inhibiting Smad2 phosphorylation and nuclear translocation, then suppressing Smad2 combined with the promoter of Snail which inhibited the transcriptional expression of Snail. These findings suggesting curcumin could be a useful agent for antitumor therapy and also a promising drug combined with other strategies to preventing and treating HCC.

## INTRODUCTION

It was reported that transforming growth factor-β1 (TGF-β1) was a potent inductor of growth in normal rat kidney fibroblast in the soft agar assay [1]. TGF-β1 has been involved in a lot of distinct biological processes, which include embryonic stem cell differentiation and self-renewal, homeostasis of differentiated cells, immune system suppression, and promotion of cancer development [2]. During the advance of malignancy, one of the main characteristics of a cancer cell is its enhanced capacity for migration, which allows invasion of surrounding tissues and metastasis to different organs [3, 4]. In epithelial-derived cancers, epithelial-mesenchymal transitions (EMT) was proved to be an important contributor for cancer invasion and metastasis [5, 6]. EMT refers to the transfer of epithelial cells to mesenchymal cells morphologically, which is a basic physiological and pathological phenomena, and plays an important role in the process of early embryogenic morphogenesis [7]. TGF-β1 was certified as a major inducer of EMT, it was reported that TGF-β1 induced EMT in Smad-independent and Smad-dependent two pathways [8]. In Smad-dependent pathway, TGF-β1 signals are started by TβR II and TβR I receptors, then transduced by Smads which as the intracellular mediators [9]. In the Smad-independent pathway, TGF-β1 signals was transduced by TβR II and TβR I receptors which interacted with the MAPK signaling pathway [10]. However, in hepatoma cells, the extrinsic signals of EMT regulated by TGF-β1 remains to be elucidated, and searching drugs to inhibit TGF-β1 induced EMT may be a very promising therapeutic strategy in hepatocellular carcinoma (HCC).

Fortunately, in the present article, we found that curcumin inhibited EMT induced by TGF-β1 in hepatoma cells. Curcumin is a single compound derived from curcuma longa, in India, it has found an wide utilization in medical purposes, and studies demonstrated that curcumin exerts many function including antioxidant, anti-inflammatory and antitumor properties *in vitro* and *in vivo* [11, 12]. Furthermore, it was demonstrated that curcumin inhibited IFN-γ-induced IDO and TNF-α-induced EMT in our previous study [13]. In this article, we elucidated the mechanism of curcumin inhibited EMT induced by TGF-β1 in hepatoma cells, which intended to provide experimental support for curcumin in preventing and treating HCC.

## RESULTS

### TGF-β1 induced EMT in hepatoma cells

**Figure 1:**
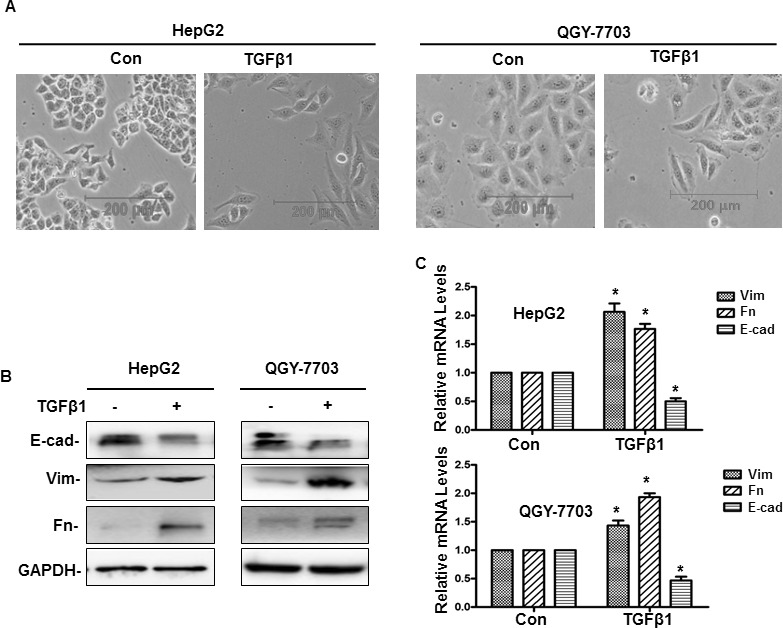
TGF-β1-induced EMT in hepatoma cells (**A**) Cells were treated with or without 20 ng/ml TGF-β1 for 48 h, and then the phenotypic changes of EMT in hepatoma cell lines were detected by a phase contrast microscope (A), the protein and mRNA expression of E-cadherin (E-cad), vimentin (Vim) and fibronectin (Fn) were detected by western blotting and quantitative real-time PCR respectively (**B**, **C**). GAPDH served as the loading control. Similar results were obtained in three independent experiments. Statistically significant values with *P* < 0.01 are marked with (^*^).

To study the influence of TGF-β1 on EMT, hepatoma cell lines treatment with or without 20 ng/ml TGF-β1. After treated with TGF-β1, phase contrast microscope demonstrated that cell lines including HepG2 and QGY-7703 adopted a typical fibroblast-like morphology of mesenchymal cells and became scattered (Figure 1A). Real-Time PCR and western blotting indicated that the expression of vimentin and fibronectin were upregulated, while E-cadherin were downregulated significantly (Figure 1B, 1C). All these results indicate that EMT can be induced by TGF-β1 in hepatoma cells.

### TGF-β1 promoted invasion and metastasis in hepatoma cells

**Figure 2:**
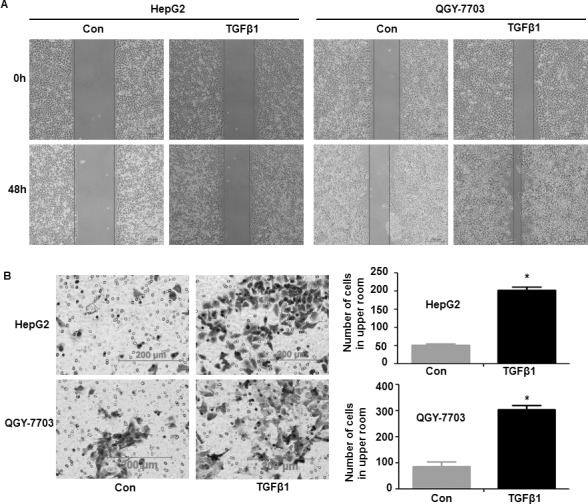
TGF-β1-induced invasion and metastasis in hepatoma cells Cells were treated with or without 20 ng/ml TGF-β1 for 48 h, and then the invasion and metastasis of hepatoma cells were detected by wound healing technology (**A**) and cell invasion assay (**B**). In cell invasion assay, cells that had spread through the pores of the filter and into the lower chamber were stained with hematoxylin and counted under a phase contrast microscope (five fields per chamber). Similar results were obtained in three independent experiments. Statistically significant values with *P* < 0.01 are marked with (^*^).

After EMT, to determine the changes of invasion and metastasis ability, hepatoma cells treatment with or without TGF-β1 for 48 h. Wound healing demonstrated that treatment with TGF-β1, the scratch wound of cells become narrower significantly compared with control (Figure 2A). Cell invasion assay shown that treatment with TGF-β1, the number of cells in the lower chamber are more than the control group (Figure 2B). Therefore, these results demonstrate that TGF-β1 can induce EMT and promote hepatoma cells invasion and metastasis.

### Curcumin inhibited EMT induced by TGF-β1 in hepatoma cells

**Figure 3:**
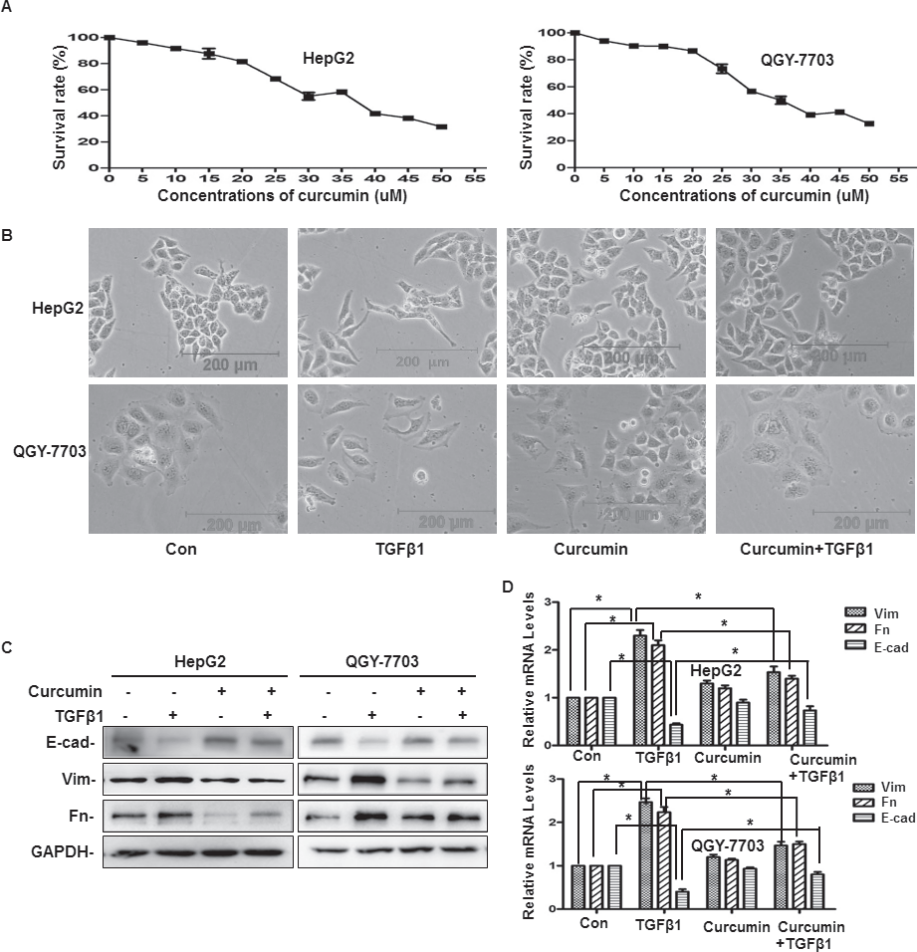
Curcumin inhibited TGF-β1-induced EMT in hepatoma cells (**A**) Cytotoxicity of curcumin in hepatoma cells were performed by means of an MTT enzyme assay. Hepatoma cells were incubated in the presence of different concentrations of curcumin at 37°C for 48 h. Each column represents the mean ± SD with respect to 100% control. At least three independent assays were performed. (**B**–**D**) Cells were pretreated with or without 20 μM curcumin for 4 h and then treated with or without 20 ng/ml TGF-β1 for 48 h, then the phenotypic changes of EMT in hepatoma cell lines were detected by a phase contrast microscope (B), the protein and mRNA expression of E-cad, Vim and Fn were detected by western blotting and quantitative real-time PCR respectively (C, D). GAPDH served as the loading control. Similar results were obtained in three independent experiments. Statistically significant values with *P* < 0.01 are marked with (^*^).

At first, an MTT enzyme assay was used to determine the cytotoxicity of curcumin in hepatoma cell lines (HepG2 and QGY-7703). It demonstrated that curcumin could suppress the proliferation of cells in a concentration-dependent manner. At 20 μM of curcumin, the survival rate of cells was about 80%, whereas when cells were exposed to higher concentration, the cells died significantly (Figure 3A). Therefore, 20 μM curcumin were used in subsequent experiments, which still maintain the high survival rate of the hepatoma cells. To evaluate the role of curcumin on TGF-β1-induced EMT, cells pretreated with or without 20 μM curcumin, then treated with or without TGF-β1 for 48 h. It demonstrated that TGF-β1-treated cells exhibited morphological changes of EMT, the expression of vimentin and fibronectin increased and the expression of E-cadherin decreased. But when pretreated with curcumin, TGF-β1-induced morphological changes could not be observed any more, and expression of vimentin, fibronectin and E-cadherin were recovered to the control group (Figure 3B–3D). This results indicate that curcumin can inhibit EMT induced by TGF-β1 in hepatoma cells.

### Curcumin inhibited hepatoma cells invasion and metastasis driven by TGF-β1

**Figure 4:**
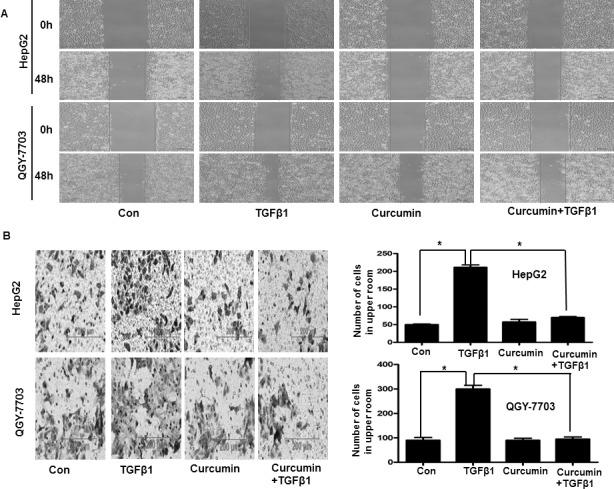
Curcumin inhibited TGF-β1-induced invasion and metastasis in hepatoma cells Cells were pretreated with or without 20 μM curcumin for 4 h and then treated with or without 20 ng/ml TGF-β1 for 48 h, then the invasion and metastasis of hepatoma cells were detected by wound healing technology (**A**) and cell invasion assay (**B**). In cell invasion assay, cells that had spread through the pores of the filter and into the lower chamber were stained with hematoxylin and counted under a phase contrast microscope (five fields per chamber). Similar results were obtained in three independent experiments. Statistically significant values with *P* < 0.01 are marked with (^*^).

To determine the influence of curcumin on hepatoma cells invasion and metastasis which driven by TGF-β1-induced EMT, after cells treatment with or without 20 μM curcumin for 4 h, then cells treatment with or without 20 ng/ml TGF-β1 for 48 h. Wound healing analysis demonstrated that when cells treated with 20 ng/ml TGF-β1, the scratch wound of cells become narrower significantly compared with control. But when cells pretreated with curcumin, the scratch wound of cells recover to control group (Figure 4A). The results of cell invasion assay revealed that when treatment with 20 ng/ml TGF-β1, the number of cells in the lower chamber are more than the control group, when pretreated with curcumin, cells count in the lower chamber recover to control group (Figure 4B). Therefore, all these findings suggest that curcumin can suppress invasion and metastasis driven by TGF-β1-induced EMT in hepatoma cells.

### Curcumin via downregulation of Snail to inhibit EMT induced by TGF-β1 in hepatoma cells

**Figure 5:**
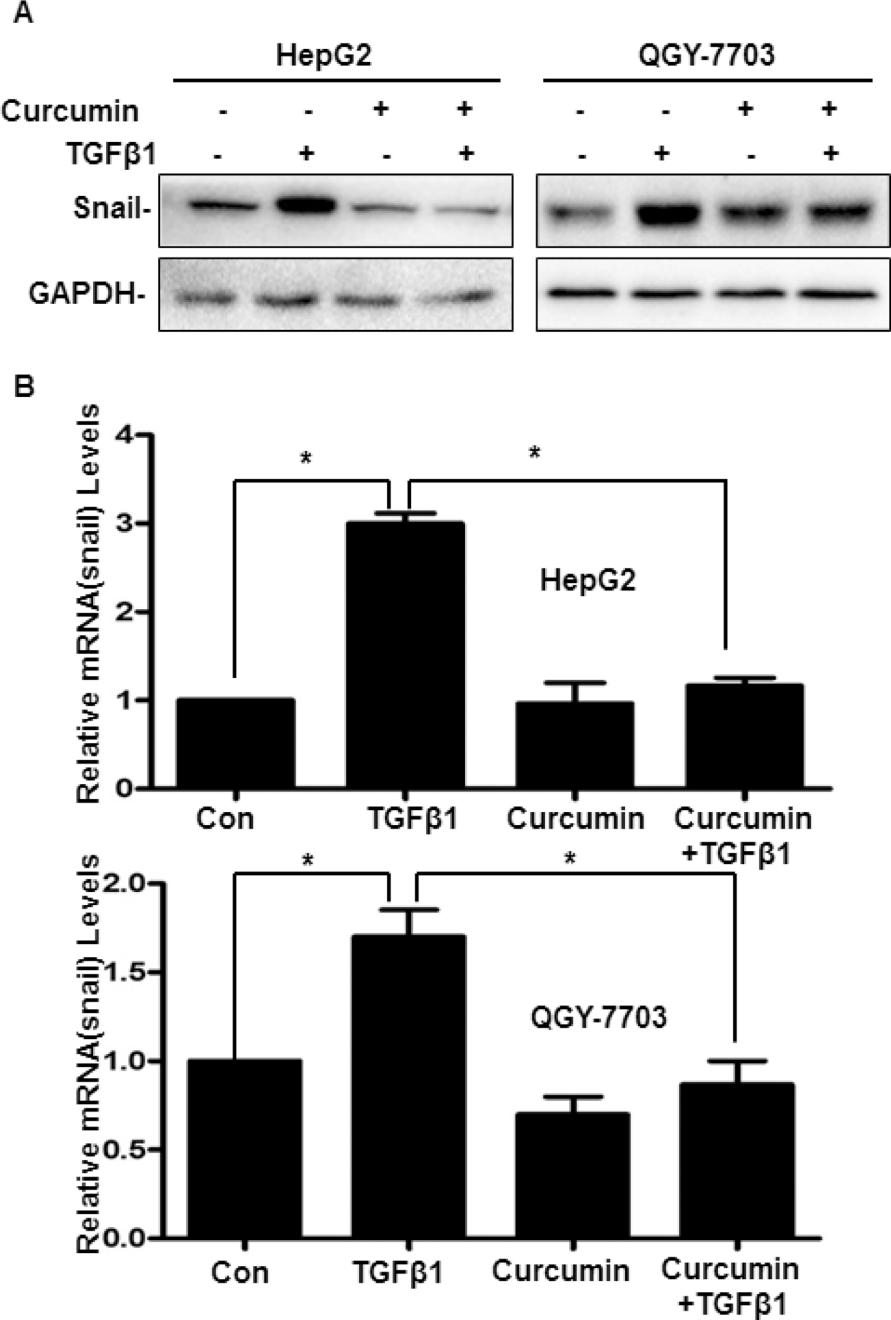
Curcumin via downregulation of Snail to inhibit TGF-β1-induced EMT in hepatoma cells Cells were pretreated with or without 20 μM curcumin for 4 h and then treated with or without 20 ng/ml TGF-β1 for 48 h, the protein and mRNA expression of Snail were detected by western blotting (**A**) and quantitative real-time PCR (**B**) respectively. GAPDH served as the loading control. Similar results were obtained in three independent experiments. Statistically significant values with *P* < 0.01 are marked with (^*^).

It is well known that as a molecular organizer, Snail is activated by most pathways triggering EMT via down-regulating the epithelial genes and up-regulating the mesenchymal genes. Therefore, to determine the mechanisms of curcumin inhibiting TGF-β1-induced EMT, firstly, cells treatment with or without 20 μM curcumin for 4 h, then treatment with or without 20 ng/ml TGF-β1 for 48 h. As shown in Figure 5, both mRNA and protein of Snail was elevated by TGF-β1 significantly, but when pretreated with curcumin, the expression of mRNA and protein of Snail induced by TGF-β1 was downregulated significantly (Figure 5). These results indicated that Snail acted as an incentive role during the process of TGF-β1-induced EMT, and curcumin via downregulation of Snail to inhibit EMT induced by TGF-β1 in hepatoma cells.

### Curcumin downregulation the expression of Snail by supressing Smad2 phosphorylation and nuclear translocation

**Figure 6:**
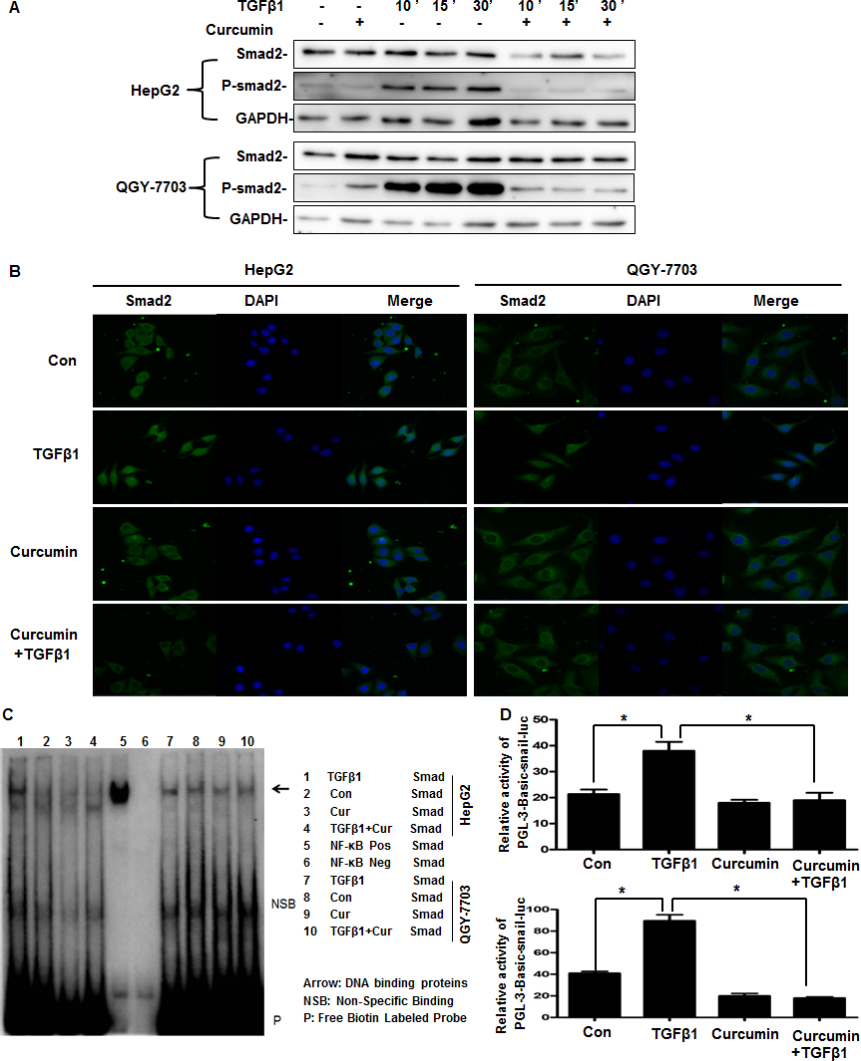
Curcumin downregulation the expression of Snail by supressing Smad2 phosphorylation and nuclear translocation (**A**) Cells were pretreated with or without 20 μM curcumin for 4 h and then treated with or without 20 ng/ml TGF-β1 for 10 min, 15 min, 30 min respectively. The phosphorylation of Smad2 and total Smad2 were detected by western blotting. GAPDH served as the loading control. Similar results were obtained in three independent experiments. (**B**) Cells were pretreated with or without 20 μM curcumin for 4 h and then treated with or without 20 ng/ml TGF-β1 for 30min, immunofluorescence and confocal microscopy detection of Smad2 were performed as described in Section 2. (**C**) Cells were pretreated with or without 20 μM curcumin for 4 h and then treated with or without 20 ng/ml TGF-β1 for 1 h, the combination of Smads and Snail promoter was detected by EMSA. (**D**) Cells were transfected with pGL3-Basic-Snail-luc reporter plasmid for 6 h, then cells were pretreated with or without 20 μM curcumin for 4 h and then treated with or without 20 ng/ml TGF-β1 for 24 h. Luminescence was measured by a luminometer. pRL-TK plasmids served as the correcting transfection efficiency. Results were expressed as the ratios between the activity of the reporter plasmid and pRL-TK. Similar results were obtained in three independent experiments. Statistically significant values with *P* < 0.01 are marked with (^*^).

It was reported that Smads combined with the binding sequence of gene promoter is CAGAA/C [14], we found that there were many concensus sequence CAGAA/C in the promoter of Snail (422–436). To determine the detailed mechanisms of HDACIs regulating Snail, we firstly studied the influence of curcumin on Smad2, which is the key mediator in TGF-β-induced EMT via Smad-dependent pathway [15]. It demonstrated that the phosphorylation of Smad2 increased after treatment with TGF-β1 for 10 min, the Smad2 phosphorylation reached the peak levels after treated with TGF-β1 for 30 min. But when pretreated with curcumin before treatment with TGF-β1, Smad2 phosphorylation was suppressed significantly (Figure 6A). These data indicated that curcumin via inhibiting the TGF-β1/Smads signaling pathway to suppress TGF-β1-induced EMT .

As a nuclear transcription factor, Smad2 requires nuclear translocation to exert its biological function. We further studied whether curcumin inhibited Smad2 nuclear translocation. Confocal results in untreated cells showed that Smad2 was localized exclusively in the cytoplasm, and curcumin had no influence on Smad2 subcellular localization. In contrast, after cells were treated with TGF-β1 for 30 minutes, Smad2 had a significant nuclear translocation from cytoplasm, but cells pretreatment with curcumin for 4 hours markedly reduced TGF-β1-induced nuclear translocation of Smad2 (Figure 6B). After the Smad2 enter into nuclear, they will combine with the gene promoter to play their biological functions. EMSA result further confirmed that TGF-β1 promoted the Smads combined with the Snail promoter, and curcumin inhibited the combination between Snail promoter and Smads which promoted by TGF-β1 (Figure 6C). Further Dual-Glo-luciferase analysis demonstrated that curcumin significantly suppressed pGL3-Basic-Snail-luc activity which enhanced by TGF-β1 in hepatoma cells (Figure 6D), which is in accord with the results that curcumin inhibited the mRNA and protein expression of Snail (Figure 5). Generally, our results indicated that curcumin downregulation the expression of Snail induced by TGF-β1 via inhibiting Smad2 phosphorylation and nuclear translocation, then suppressing the combination with the promoter of Snail to inhibit the transcription of Snail, which will inhibit the TGF-β1-induced EMT in hepatoma cells.

## DISCUSSION

HCC is one type of liver malignancy, and it is the most common type of cancer in human beings [16]. Although in certain types of cancer, the incidence has been declined, HCC has been increasing worldwide day by day [17, 18]. At present, Resection and transplantation are still the available curative treatments, but which are hampered because of their high recurrence rates and development of metastasis [19, 20]. EMT was identified as the important initiation step for cancer metastasis [21]. When EMT occurred, E-cadherin was down-regulated and vimentin was up-regulated, they were regarded as the important markers of EMT [22–24]. Accumulating data suggested that EMT played the key role in the process of HCC progression and metastasis [25, 26]. Therefore, to reduce the morbidity and mortality rates of HCC, the molecular targets in the pathway of EMT have received great attention, and more effective interventions may provide potential strategies for HCC treatment.

TGF-β1 was identified as a critical modulator for EMT in many types of cancers including HCC [27, 28]. Many studies demonstrate that TGF-β1 is a major inducer of EMT which promotes cancer progression, and this function has been well established in experimental models in HCC [29–31]. Just because of these, TGF-β1 is thought to be an important factor regulating the development and progression in HCC. Therefore, searching drugs to inhibit TGF-β1 induced EMT maybe a promising strategy in treatment of HCC.

It was reported that as a potent anti-inflammatory agent, curcumin also has strong therapeutic potential for many types of cancers [32]. A recent study demonstrated that curcumin inhibits proliferation and promotes apoptosis of human HCC cells via suppressing the Wnt signaling pathway [33]. It was also reported that in HepG2 cells, inhibiting HIF-1α stabilization was found to be a mechanism for explaining the antitumor effect of curcumin [34]. Furthermore, in our past study, we demonstrated that curcumin could inhibit EMT induced by TNFα, when it combined with antitumor vaccine which had significant effect on treatment of cancer [13]. In this article, it shown that curcumin could inhibit EMT induced by TGF-β1 in hepatoma cells. Our study provides several insights into curcumin regulating EMT and metastasis induced by TGF-β1. First, our study demonstrated that TGF-β1 made hepatoma cells became mesenchymal cells and adopted a fibroblast-like morphology, and upregulated vimentin and fibronectin, downregulated E-cadherin significantly, which indicated that TGF-β1 could induce hepatoma cells occurs EMT (Figure 1). Furthermore, wound healing and cell invasion assays demonstrated that TGF-β1 induced-EMT could triggered hepatoma cells’ invasion and metastasis (Figure 2).

Second, our study reveals that curcumin inhibited TGF-β1-induced EMT, invasion and metastasis in hepatoma cells. We found that after pretreated with curcumin, TGF-β1-induced morphological changes of EMT was inhibited, decreased vimentin and fibronectin levels which increased by TGF-β1 and increased E-cadherin levels which decreased by TGF-β1 (Figure 3). Further wound healing and cell invasion assays all demonstrated that curcumin could inhibit invasion and metastasis which driven by TGF-β1-induced EMT (Figure 4). Therefore, all these findings indicate that curcumin can suppress hepatoma cells’ invasion and metastasis which driven by TGF-β1-induced EMT.

Third, our results demonstrated that curcumin mainly via inhibiting the expression of Snail to inhibited TGF-β1-induced EMT. Snail, a zinc-finger transcription factor, is an important factor in regulating EMT and has a crucial function in tumor progression by facilitating tumor cell migration and invasion [35, 36]. In the present article, it demonstrated that curcumin inhibited Snail expression which induced by TGF-β1 via inhibiting Smad2 phosphorylation and nuclear translocation, then suppressing Smad2 combined with the promoter of Snail which inhibited Snail transcription (Figures 5, 6). The current study demonstrates the detailed molecular mechanism of curcumin inhibits hepatoma cells’ invasion and metastasis which driven by TGF-β1-induced EMT, all these results suggesting curcumin is a potential anticancer agent and also a promising drug combined with other strategies to preventing and treating HCC.

## MATERIALS AND METHODS

### Chemicals and reagents

Curcumin was purchased from Sigma–Aldrich (Deisenhofen, Germany). pGL3-Snail was a gift from Sun Yat-sen University, pRL-TK and dual-luciferase assay kit are products of Promega (Madison, WI, USA). The monoclonal anti-Snail, an-Smad2, anti-p-Smad2, anti-E-cadherin, anti-fibronectin, anti-GAPDH, anti-Vimentin antibody, and the secondary anti-mouse antibody conjugated to HRP are products of Cell Signaling Technology (MA, USA). Millicell chamber (8 μm) was purchased from Millipore (BD Biosciences, USA). SYBR Premix Ex Taq II is a product of TaKaRa BIO Inc (TBI, Japan). The secondary anti-mouse antibody conjugated to FITC and DAPI dye were purchased from Invitrogen (Carlsbad, CA, USA).

### Cell culture

The human hepatoma cell lines HepG2 and QGY-7703 were purchased from ATCC. The two cell lines was used from July 2015 to October 2016. Both cell lines were authenticated by short tandem repeat analysis and passaged for fewer than 6 months before experiments. Vials were thawed and maintained in culture for only several weeks at a time. Cells were maintained in DMEM supplemented with 10% heat-inactivated endotoxin-free Newborn Calf Serum, 100 µg/ml streptomycin and 100 units/ml penicillin under a humidified 5% CO_2_ atmosphere at 37°C in a incubator.

### Cytotoxicity assay

The cytotoxicity of curcumin toward the cultured cells was assessed using MTT [3-(4,5-dimethylthiazol-2yl)-2,5-diphenyltetrazolium bromide] assays (Sigma Chemical Co). Cells were seeded onto 96-well microplates at a density of 1 × 10^4^ cells per well and incubated for 24 hours. Cells were then treated with selected concentrations of SAHA and NaB for 24 hours. Cells in culture medium alone served as the untreated control. The MTT reagent (5 mg/mL in distilled water) was prepared immediately prior to use. After removing the incubation medium from the wells, cells were washed with PBS, and 10 mL of MTT reagent was added. After incubation for 4 hours at 37°C, MTT reagent in 100 mL of dimethylsulfoxide (DMSO) was added to each well. Surviving cells were then detected by measuring absorbance at 570 nm using a plate reader. The cell viability was expressed as a percentage of the values obtained for the controls.

### Quantitative real-time PCR

Cells (2 × 10^5^) were plated on 6-well plates, after treatment with drugs, cells were washed twice with ice-cold PBS. Total mRNA was extracted with TRIZOL reagent. The first strand of cDNA was generated from 2 μg total RNA using oligo-dT primer and Superscript II Reverse Transcriptase (GIBCO BRL, Grand Island, NY, USA). Quantitative Real-Time PCR was run on an iCycler (Bio-rad, Hercules, CA, USA) using validated primers and SYBR Premix Ex Taq II (Takara, Japan) for detection. The cycle number when the fluorescence first reaches a preset threshold (Ct), allow the quantification of the specific template concentration. Transcripts of the housekeeping gene GAPDH in the same incubations were used for internal normalization.

### Western blotting analysis

Cells were lysed in cell lysis buffer, lysates were cleared by centrifugation and denatured by boiling in Laemmli buffer. Equal amounts of protein samples were separated on 12% sodium dodecyl sulfate (SDS)–polyacrylamide gels and electrophoretically transferred to nitrocellulose membranes. Following blocking with 5% non-fat milk at roomtemperature for 2 h, membranes were incubated with the primary antibody at 1:1000 dilution overnight at 4°C and then incubated with a horseradish peroxidase-conjugated secondary antibody at 1:5000 dilution for 1 h at room temperature. Specific immune complexes were detected using western blotting Plus Chemiluminescence Reagent (Life Science, Inc., Boston, MA, USA).

### Wound healing

HepG2 and QGY-7703 cells (4 × 10^5^) were cultured on 6-well plates. After 24 h of cultivation, cells covered the whole bottom of the well. A defined scratch was applied on the well bottom, which detached cells within a definite corridor. Cells pretreated with or without curcumin for 4 h, then treated with or without TGF-β1 for 48 h, the percentage recovering of the scratch wound was monitored.

### Cell invasion assay

The cell invasion assay was performed as follows: first, 6.5-mm-diameter polycarbonate filters (8 μm pore size) were coated with Matrigel TM, dried, and reconstituted at 37°C with appropriate DMEM before use. Then 1 × 10^5^ HepG2 or QGY-7703 cells per chamber were added to the upper chamber in DMEM containing 1% FCS, while lower chamber in DMEM containing 10% FCS. pretreated with or without curcumin for 4 h, then treated with or without TGF-β1 for 48 h, the drug concentration was the same in the lower and upper chambers. The number of cells that had spread through the pores of the filter and into the lower chamber was counted under a phase contrast microscope (five fields per chamber). Each invasion experiment was carried out in duplicate and repeated in three independent experiments.

### Confocal microscopy for Smad2

Cells were grown on chamber slides. After 12 h of cultivation, pretreated with or without curcumin for 4 h, then treated with or without TGF-β1 for 30 min. Cells were fixed in 4% paraformaldehyde for 30 min and blocked with goat serum for 30 min at 37°C, then incubated with anti-Smad2 antibody at 1:100 for 1 h at 37°C. Slides were washed with PBS and incubated with a secondary anti-mouse antibody conjugated to FITC at 1:1000 for 45 min at 37°C. After washed by PBS, the cells were incubated with DAPI (10 μg/ml) for 10 min to visualize cell nuclei. Samples were examined with Confocal Laser Scanning Microscopy (Zeiss, Germany) to analyze nuclear translocation of Smad2.

### Electrophoresis mobility shift assay (EMSA)

Nuclear extracts from cells pretreated with or without curcumin for 1 h, then treated with or without TGF-β1 for 1 h were used in EMSA with an oligonucleotide probe wich is the sequence from Snail promotor containing the Smads binding site (CAGAA/C). The coding strand of the oligonucleotide probe is 5′-CCAGGGGGCGTCAGAAGCGCTCAGACCACC GGGCGC-3′, Antibodies used for super-shifting the complex were purchased from Santa Cruz (Santa Cruz, CA, USA).

### Transient transfection and reporter genes assay

To measure the transcriptional activity of Snail, cells were transfected with 0.2 µg DNA/cm2 per pGL3-Snail plasmid and lipofectamine 3000 reagent (Invitrogen, USA) according to the manufacturer’s instructions. Transfection efficiency was normalized by cotransfection with pRL-TK. Transcriptional activity was determined by a luminometer, using a dual-luciferase assay kit. Results were displayed as the ratios between the activity of the reporter plasmid and pRL-TK.

### Statistical analyses

All values were reported as mean ± SEM of three independent experiments unless otherwise specified. Data were analyzed by two-tailed unpaired Student’s t-test between two groups and by One-Way ANOVA followed by Bonferroni test for multiple comparison involved. These analyses were performed using GraphPad Prism Software Version 5.0 (GraphPad Software Inc., La Jolla, CA, USA). *P* < 0.05 was considered statistically significant.
